# Pairwise statistical significance of local sequence alignment using multiple parameter sets and empirical justification of parameter set change penalty

**DOI:** 10.1186/1471-2105-10-S3-S1

**Published:** 2009-03-19

**Authors:** Ankit Agrawal, Xiaoqiu Huang

**Affiliations:** 1 Department of Computer Science, Iowa State University, 226 Atanasoff Hall, Ames, IA 50011-1041, USA

## Abstract

**Background:**

Accurate estimation of statistical significance of a pairwise alignment is an important problem in sequence comparison. Recently, a comparative study of pairwise statistical significance with database statistical significance was conducted. In this paper, we extend the earlier work on pairwise statistical significance by incorporating with it the use of multiple parameter sets.

**Results:**

Results for a knowledge discovery application of homology detection reveal that using multiple parameter sets for pairwise statistical significance estimates gives better coverage than using a single parameter set, at least at some error levels. Further, the results of pairwise statistical significance using multiple parameter sets are shown to be significantly better than database statistical significance estimates reported by BLAST and PSI-BLAST, and comparable and at times significantly better than SSEARCH. Using non-zero parameter set change penalty values give better performance than zero penalty.

**Conclusion:**

The fact that the homology detection performance does not degrade when using multiple parameter sets is a strong evidence for the validity of the assumption that the alignment score distribution follows an extreme value distribution even when using multiple parameter sets. Parameter set change penalty is a useful parameter for alignment using multiple parameter sets. Pairwise statistical significance using multiple parameter sets can be effectively used to determine the relatedness of a (or a few) pair(s) of sequences without performing a time-consuming database search.

## Background

Local sequence alignment plays a major role in the analysis of DNA and protein sequences [1-3]. It is the basic step of many other applications like detecting homology, finding protein structure and function, deciphering evolutionary relationships, etc. There exist several local sequence alignment programs that use well-known algorithms [4,5] or their heuristic versions [3,6,7]. Database search is a special case of pairwise local sequence alignment where the second sequence is a database in itself consisting of many sequences. Recently, there have been many enhancements in alignment program features [8,9] using difference blocks and multiple scoring matrices, in an attempt to incorporate more biological features in the alignment algorithm.

### Why statistical significance?

The local sequence alignment programs report alignment scores for the alignments constructed, and related (homologous) sequences will have *higher *alignment scores. But the definition of *high *depends strongly on the alignment score distribution, which gives importance to the concept of statistical significance. An alignment score is considered statistically significant if it has a low probability of occurring by chance. Since the alignment score distribution depends on various factors like alignment program, scoring scheme, sequence lengths, sequence compositions [10], it implies that it is possible to have two alignments of different sequence pairs with scores *x *and *y *with *x *<*y*, but *x *more significant than *y*. Therefore, instead of using the alignment score for detecting homology, the statistical significance of an alignment score is more widely accepted as a metric to comment on the relatedness of the two sequences being aligned. Of course, it is important to emphasize here that although statistical significance is a good preliminary indicator of biological significance, it does not necessarily imply biological significance [10,11].

Accurate statistical theory for the ungapped alignment score distribution is available [12]. However, no precise statistical theory currently exists for the gapped alignment score distribution and for score distributions from alignment programs using additional features. Accurate estimation of statistical significance of gapped sequence alignment scores has attracted a lot of attention in the recent years [13-21].

Although there exists some understanding of the statistics of gapped alignment score distributions for simple scoring schemes [22,23], but a complete mathematical description of the optimal score distribution remains far from reach [23]. There exist some excellent reviews on statistical significance in sequence comparison in the literature [10,24-26].

### Database statistical significance versus pairwise statistical significance

Recently, a study of pairwise statistical significance and its comparison with database statistical significance [27] was conducted. In summary, the database statistical significance reported by most database search programs like SSEARCH, FASTA, PSI-BLAST is database-dependent, and hence, the same alignment of two sequences with the same alignment score can be evaluated as having different significance values in database searches with different databases, and even with the same database at different times, as the database size can be variable. On the other hand, pairwise statistical significance is specific to the sequence pair being aligned, and is database-independent. In [27], various approaches to estimate pairwise statistical significance were compared to find that maximum likelihood fitting with censoring left of peak is the most accurate method for estimating pairwise statistical significance. Further, this method was compared with database statistical significance in a homology detection experiment to find that pairwise statistical significance performs comparably to and sometimes significantly better than database statistical significance.

Accurate statistical significance estimates for pairwise alignments can be very useful to comment on the relatedness of a pair of sequences aligned by an alignment program independent of any database. And thus, it can also be used to compare different combination of alignment parameters – like the alignment program itself, substitution matrices, gap costs. A comparison of different gap opening penalties for four commonly used BLOSUM matrices using pairwise statistical significance was presented in [28]. In addition to the standard local alignment algorithms [4,5], some recent algorithms have been developed [8,9] that take into account other desirable biological features in addition to gaps – like difference blocks or the use of multiple parameter sets (substitution matrices, gap penalties). These features of the alignment programs enhance the sequence alignment of real sequences by better suiting to different conservation rates at different spatial locations of the sequences. As pointed out earlier, accurate statistical theory for alignment score distribution is available only for ungapped alignment, and not even for its simplest extension, i.e., alignment with gaps. Accurate statistics of the alignment score distribution from newer and more sophisticated alignment programs therefore is not expected to be straightforward. For comparing the performance of newer alignment programs, accurate estimates of pairwise statistical significance can be very useful. Further, quick and accurate estimates of pairwise statistical significance can also be helpful for applications like multiple sequence alignment and phylogenetic tree construction which are based on pairwise sequence alignment to select most related pairs of sequences, for example, in a progressive multiple sequence alignment.

### The extreme value distribution for ungapped and gapped alignments

Just as the distribution of the sum of a large number of independent identically distributed (i.i.d.) random variables tends to a normal distribution (central limit theorem), the distribution of the maximum of a large number of i.i.d. random variables tends to an extreme value distribution (EVD) [29]. This is an important and useful fact, because in principle it allows us to fit an EVD to the score distribution from any local alignment program and use it for estimating statistical significance of scores from that program. The distribution of Smith-Waterman local alignment score between random, unrelated sequences is approximately a Gumbel-type EVD [12]. In the limit of sufficiently large sequence lengths *m *and *n*, the statistics of HSP (High-scoring Segment Pairs which correspond to ungapped local alignments) scores are characterized by two parameters, *K *and *λ*. The probability (P-value) that the optimal local alignment score *S *exceeds *x *is estimated by:

Pr(*S *> *x*) ~1 - *e*^-*E*^,

where *E *is the E-value and is given by

*E *= *Kmne*^-*λx*^.

For E-values less than 0.01, both E-value and P-values are very close to each other. The above formulae are valid for ungapped alignments [12], and the parameters *K *and *λ *can be computed analytically from the substitution scores and sequence compositions. For gapped alignments, no perfect statistical theory has yet been developed, although there exist some good starting points for the problem as mentioned before [22,23]. Recently, researchers have also looked closely at the low probability tail distribution, and the work in [30] applied a rare-event sampling technique earlier used in [31] and suggested a Gaussian correction to the Gumbel distribution to better describe the rare event tail, resulting in a considerable change in the reported significance values. However, for most practical purposes, the original Gumbel distribution has been widely used to describe gapped alignment score distribution [9,13-15,17,27,32,33]. From an empirically generated score distribution, we can directly observe the E-value *E *for a particular score *x*, by counting the number of times a score *x *or higher was attained. Since this number would be different for different number of random shuffles *N *(or number of sequences in the database in case of database search), a normalized E-value is defined as

$$E_{normalized}=\frac{E}{N}$$

In theory, this normalized E-value is same as the P-value (for large *N*).

## Contributions

In this paper, we extend the existing work on pairwise statistical significance [27] to incorporate in it the use of multiple parameter sets, and evaluate it on an important knowledge discovery application-homology detection. We conducted similar experiments as reported in [34], and later in [27] on a subset of the CATH 2.3 database to compare pairwise statistical significance with single and multiple parameter sets. This benchmark database was earlier created in [34] to evaluate seven protein structure comparison methods and two sequence comparison programs: SSEARCH and PSI-BLAST. SSEARCH uses the original Smith-Waterman algorithm [4], and is considered the most sensitive algorithm in terms of retrieval accuracy, better than the heuristic versions like BLAST and FASTA [35,36]. PSI-BLAST is a modification to the BLAST program, where position specific scoring matrices are constructed over multiple iterations of BLAST algorithm. Comparison of pairwise statistical significance results using multiple parameter sets with pairwise statistical significance using a single parameter set shows that at least for some error levels, using multiple parameter sets is significantly better than using a single parameter set. This is because sequences can have different conservation rates at different spatial locations, which can be better aligned using multiple parameter sets (substitution matrices, gap penalties, etc.). Comparison with database statistical significance results show that pairwise statistical significance with multiple parameter sets gives significantly better performance than the statistical significance estimates reported by BLAST and PSI-BLAST, and comparable and at times significantly better performance than the SSEARCH program. Further, the results also give concrete evidence that for the practical application of homology detection, the score distribution from alignment program using multiple parameter sets can also be assumed to follow an extreme value distribution. This is an important and useful finding since it is in general difficult to accurately determine statistics of alignment scores from enhanced alignment programs. Finally, experiments with different values of parameter set change penalties indicate that it is indeed important to use a non-zero parameter set change penalty while performing alignment using multiple parameter sets.

## Methods

### Pairwise statistical significance estimation

Consider the pairwise statistical significance described in [27] to be obtainable by the following function: *PairwiseStatSig*(*Seq*1, *Seq*2, *SC*, *N*) where *Seq*1 is the first sequence, *Seq*2 is the second sequence, *SC *is the scoring scheme (substitution matrix, gap penalties), and *N *is the number of shuffles. The function PairwiseStatSig, therefore generates a score distribution by aligning *Seq*1 with *N *shuffled versions of *Seq*2, fits the distribution to an extreme value distribution using censored maximum likelihood fitting to obtain the statistical parameters *K *and *λ*, and returns the pairwise statistical significance estimate of the pairwise alignment score between *Seq*1 and *Seq*2 using the parameters *K *and *λ *in the P-value formula. More details on pairwise statistical significance can be found in [27]. In this paper, we dynamically use multiple parameter sets instead of a single scoring scheme *SC *for estimation of pairwise statistical significance.

### Dynamic use of multiple parameter sets in sequence alignment

Usually, pairwise sequence alignment is done with a single parameter set (substitution matrix, gap penalties). But to suit the different levels of conservation between sequences, there exists an algorithm [9] which can dynamically use multiple parameter sets and generate a single optimal alignment with possibly different parameter sets used in different regions of the alignment. The algorithm is implemented in a program named GAP4. The algorithm uses a dynamic programming approach as explained in [9]. Consider alignment of two sequences *A *= *a*_1_, *a*_2_,..., *a*_*m *_and *B *= *b*_1_, *b*_2_,... *b*_*n *_using *p *parameter sets *P*_1_, *P*_2_,..., *P*_*p*_. Let *A*_*i *_and *B*_*j *_be the subsequences *a*_1_, *a*_2_,... *a*_*i *_and *b*_1_, *b*_2_,..., *b*_*j *_respectively. For each alignment position (*i*, *j*) and each parameter set *P*_*k*_, the algorithm keeps track of the optimal alignment score of the subsequences *A*_*i *_and *B*_*j *_where the last component (substitution, gap, or difference block) is scored using *P*_*k*_. Dynamic programming is used to get optimal alignment for progressive alignment positions, until *i *becomes *m *and *j *becomes *n*. Appropriate modification of the algorithm also allows it to calculate the optimal local alignment. More details about using multiple parameter sets for pairwise sequence alignment can be found in [9].

### Evaluation methodology

To evaluate the performance of pairwise statistical significance using multiple parameter sets, we used a non-redundant subset of the CATH 2.3 database (Class, Architecture, Topology, and Hierarchy, [37]) provided by [34] and available at . This database was selected in [34] to evaluate seven structure comparison programs and two sequence comparison programs. As described in [34], this dataset consists of 2771 domain sequences and includes 86 selected test query sequences. This domain set is considered as a valid benchmark for testing protein comparison algorithms [38].

We used this database and query set for experimenting with pairwise statistical significance using multiple parameter sets. For each of the 86 × 2771 comparisons, we used the maximum likelihood method with censoring left of peak with 1000 shuffles to fit the score distribution from the GAP4 program with substitution matrices BLOSUM45, BLOSUM50, BLOSUM62, BLOSUM80, and their all possible combinations (2^4 ^- 1 = 15 in number). All matrices were used in 1/3 bit scale. The gap opening penalties for each of these matrices was set to the values empirically determined to be the best for this database in [28]. These are listed in Table 1. The gap extension penalties were set to 2 for all the four matrices. Following [27,34], Error per Query (EPQ) versus Coverage plots were used to present the results. To create these plots, the list of pairwise comparisons was sorted based on decreasing statistical significance (increasing P-values). Going down the list, the coverage count is increased by one if the two sequences of the pair are homologs, and the error count is increased by one if they are not. At a given point in the list, Errors Per Query (EPQ) is the total number of errors incurred so far, divided by the number of queries; and coverage is the fraction of total homolog pairs so far detected. In the ideal case, the curve would go from 0% to 100% coverage, without incurring any errors, which would correspond to a straight line on the x-axis. Therefore, the more the curve is towards the right, the better the curve is.

**Table 1:** Effective gap opening penalties for commonly used BLOSUM matrices determined for the benchmark database used

**BLOSUM matrix**	**Gap opening penalty**
BLOSUM45	7
BLOSUM50	9
BLOSUM62	11
BLOSUM80	13

Just as gap opening and gap extension penalties are dynamically charged during the alignment process whenever a gap is inserted and extended respectively, the GAP4 [9] program allows the use of a parameter set change penalty, which is dynamically charged whenever the parameter set mapping is changed during the alignment process. To see the effect of parameter set change penalty on the coverage performance, we conducted a series of homology detection experiments with one of the substitution matrix combinations (BLOSUM45 and BLOSUM62) with different parameter set change penalties. Coverage vs. parameter set change penalty curves were plotted at different error levels to find the usefulness of the parameter set change penalty, as reported in the next section.

## Results

### Comparison with pairwise statistical significance using single parameter set

**Figure 1:**
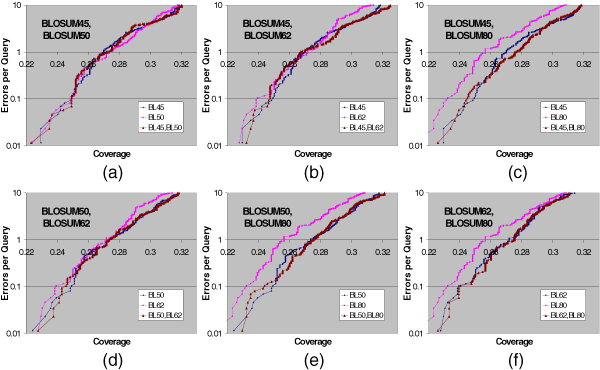
**Pairwise statistical significance using two parameter sets**. Errors per Query vs. Coverage plot for pairwise statistical significance using two parameter sets, along with the curves using corresponding single parameter sets. (a) BLOSUM45, BLOSUM50; (b) BLOSUM45, BLOSUM62; (c) BLOSUM45, BLOSUM80; (d) BLOSUM50, BLOSUM62; (e) BLOSUM50, BLOSUM80; (f) BLOSUM62, BLOSUM80. In 5 panels (b) through (f) out of 6, using two parameter sets leads to better coverage than using a single parameter set at most error levels.

**Figure 2:**
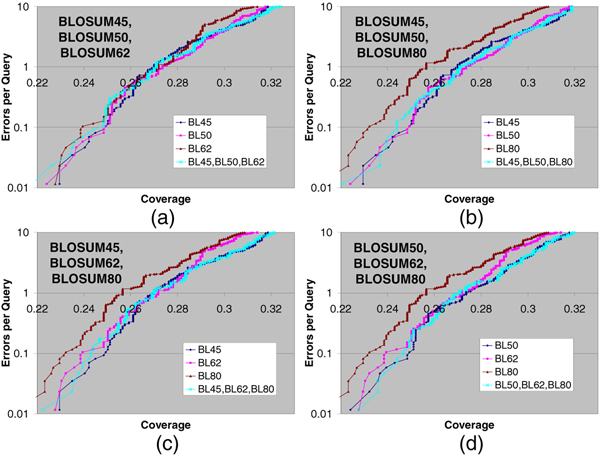
**Pairwise statistical significance using three parameter sets**. Errors per Query vs. Coverage plot for pairwise statistical significance using three parameter sets, along with the curves using corresponding single parameter sets. (a) BLOSUM45, BLOSUM50, BLOSUM62; (b) BLOSUM45, BLOSUM50, BLOSUM80; (c) BLOSUM45, BLOSUM62, BLOSUM80; (d) BLOSUM50, BLOSUM62, BLOSUM80. In all 4 panels, using three parameter sets leads to better coverage than using a single parameter set at most error levels for at least two instances of using a single parameter set.

**Figure 3:**
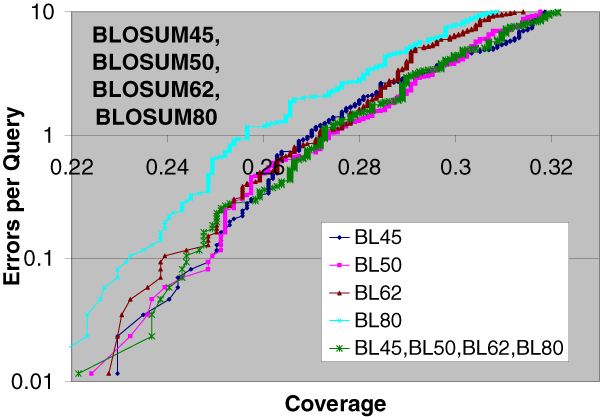
**Pairwise statistical significance using four parameter sets**. Errors per Query vs. Coverage plot for pairwise statistical significance using four parameter sets BLOSUM45, BLOSUM50, BLOSUM62, BLOSUM80 along with the curves using corresponding single parameter sets. Using four parameter sets results in better coverage than using a single parameter set at most error levels for at least three instances of using a single parameter set.

Out of the 15 substitution matrix combinations, 4 are using single parameter sets, 6 are using two parameter sets, 4 are using three parameter sets, and 1 is using all four parameter sets. The EPQ vs. Coverage curves using pairwise statistical significance with two, three, and four parameter sets are presented in Figures 1, 2, and 3 respectively. For comparison purposes, the EPQ vs. Coverage curves using corresponding single parameter sets are also presented in the same figures. The y-axis (error-axis) in all these graphs is in log-scale, and hence there is more information in the upper part of the graphs. These figures suggest that pairwise statistical significance using multiple parameter sets performs comparably to and sometimes significantly better than pairwise statistical significance using a single parameter set for most instances of using a single parameter set, and at most error levels.

### Comparison with database statistical significance

**Figure 4:**
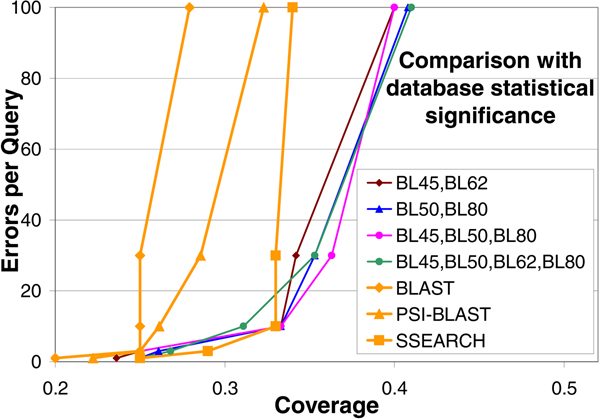
**Comparison with database statistical significance**. Comparison of pairwise statistical significance (using multiple parameter sets) and database statistical significance. All parameter combinations are significantly better than database statistical significance estimates reported by BLAST and PSI-BLAST at all error levels, and better than SSEARCH especially at higher error levels.

Since the EPQ vs. Coverage curves on the complete dataset can be distorted due to poor performance by one or two queries (if those queries produce many errors at low coverage levels) [34], to compare the performance of pairwise statistical significance using multiple parameter sets with database statistical significance, we examined the performance of the methods with individual queries, following the work in [34]. The coverage of each of the 86 queries at the 1st, 3rd, 10th, 30th, and 100th error was recorded, and the median coverage for each error level across the 86 queries was plotted to obtain EPQ vs. Coverage curves for the sequence comparison method to be evaluated. Figure 4 shows the median coverage level at the 1st, 3rd, 10th, 30th, and 100th false positive for homologs (i.e. 43 of the queries have worse coverage, and 43 have better coverage). The curve for SSEARCH in Figure 4 is derived from the figure 2A in [34]. The curves for BLAST and PSI-BLAST were obtained by experimentation. It is clear that the proposed pairwise statistical significance using multiple parameter sets performs significantly better than BLAST and PSI-BLAST at all error levels, comparable to SSEARCH at low error levels, and significantly better than SSEARCH at higher error levels.

### Empirical justification of parameter set change penalty

**Figure 5:**
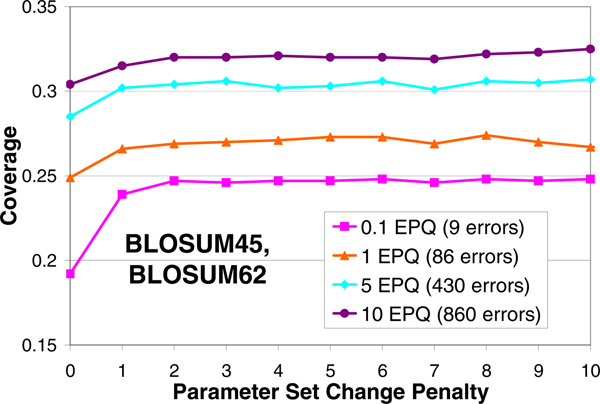
**Empirical justification of parameter set change penalty**. Coverage vs. Parameter Set Change Penalty plots at different errors per query for the substitution matrix combination of BLOSUM45 and BLOSUM62. Poor coverage is obtained if the parameter set change penalty is zero. The coverage is better and steady for non-zero values of parameter set change penalty.

The coverage vs. parameter set change penalty plot for the substitution matrix combination of BLOSUM45 and BLOSUM62 is illustrated in Figure 5. The curve shows a poor coverage performance for the case when the parameter set change penalty is not charged, i.e., when the alignment algorithm is freely allowed to change the parameter set during alignment without charging any penalty. This can be explained by the fact that the algorithm would try to mathematically maximize the alignment score by changing the parameter set as frequently as possible, which may produce more biologically irrelevant alignments. A similar phenomenon is also observed when very low gap penalty is used [28]. The coverage performance clearly improves for non-zero values of parameter set change penalty, which provides its empirical justification.

## Discussion

As pointed out earlier, SSEARCH employs the original Smith-Waterman algorithm for alignment, and is considered more sensitive than its heuristic implementations like BLAST and FASTA. PSI-BLAST uses an iterative approach with query-specific substitution matrices, and its performance mainly depends on the quality of position-specific scoring matrices (PSSMs) constructed iteratively. The results show that PSI-BLAST gave poorer performance than pairwise statistical significance using multiple parameter sets, even with PSSMs constructed against the benchmark CATH database used in our experiments. However, using PSSMs derived against BLAST non-redundant protein database has been shown to give better results [34] as it uses much more information than just a pair of sequences. Comparable and at times significantly better results than SSEARCH using pairwise statistical significance with multiple parameter sets implies that statistical significance estimates at least as good as database statistical significance can be obtained by pairwise statistical significance using multiple parameter sets without having to do a time-consuming database search. This can be very useful to estimate accurate pairwise statistical significance of two (or a few) sequences, which is a common scenario in many pairwise alignment based applications like phylogenetic tree construction, progressive multiple sequence alignment.

It is important to note that the proposed method is not a database search method but statistical significance estimation method for pairwise local alignment, and the comparison with database search programs like BLAST, SSEARCH, and PSI-BLAST is of their statistical significance estimation strategies. The proposed method, as of now is not scalable to a database search, but can be used to refine the results from a fast database search program like BLAST.

Since pairwise alignment using multiple parameter sets takes more computational time than using a single parameter set, pairwise statistical significance estimation using multiple parameter sets also takes more time than pairwise statistical significance estimation using a single parameter set. In general, using *k *parameter sets increases the computation time by a factor little more than *k*. Therefore, faster methods for significance estimation can be very helpful.

## Conclusion

This paper extends the work on pairwise statistical significance by incorporating in it the use of multiple parameter sets (substitution matrices, gap penalties, etc.), and compares it with database statistical significance for the knowledge discovery application of homology detection. The results show that pairwise statistical significance using multiple parameter sets performs better than pairwise statistical significance using a single parameter set. It also performs significantly better than database statistical significance using BLAST and PSI-BLAST, and comparable and at times significantly better than database statistical significance using SSEARCH. Further, an empirical justification of the use of parameter set change penalty is provided.

Since PSI-BLAST results can be improved by using better quality PSSMs derived from larger protein databases, we believe that the performance of pairwise statistical significance can also be improved using sequence-specific/position-specific substitution matrices, which is a significant part of our future work. Another important contribution can be to estimate the pairwise statistical significance accurately in less time, since using multiple parameter sets increases the significance estimation time. Faster methods for determining pairwise statistical significance would be very useful.

## Competing interests

The authors declare that they have no competing interests.

## Authors' contributions

AA conceived the study based on the initial idea given by XH. Both AA and XH did the programming, AA carried out the experiments and analysis, and drafted the initial manuscript. Both AA and XH read and approved the final version of the manuscript.

## References

[B1] Pearson WR, Lipman DJ (1988). Improved Tools for Biological Sequence Comparison. Proc Natl Acad Sci U S A.

[B2] Altschul SF, Gish W, Miller W, Myers EW, Lipman DJ (1990). Basic Local Alignment Search Tool. Journal of Molecular Biology.

[B3] Altschul SF, Madden TL, Schäffer AA, Zhang J, Zhang Z, Miller W, Lipman DJ (1997). Gapped BLAST and PSI-BLAST: A New Generation of Protein Database Search Programs. Nucleic Acids Research.

[B4] Smith TF, Waterman MS (1981). Identification of Common Molecular Subsequences. Journal of Molecular Biology.

[B5] Sellers PH (1984). Pattern Recognition in Genetic Sequences by Mismatch Density. Bulletin of Mathematical Biology.

[B6] Pearson WR (1996). Effective Protein Sequence Comparison. Methods in Enzymology.

[B7] Pearson WR (2000). Flexible Sequence Similarity Searching with the FASTA3 Program Package. Methods in Molecular Biology.

[B8] Huang X, Chao KM (2003). A Generalized Global Alignment Algorithm. Bioinformatics.

[B9] Huang X, Brutlag DL (2007). Dynamic Use of Multiple Parameter Sets in Sequence Alignment. Nucleic Acids Research.

[B10] Mott R (2005). Alignment: Statistical Significance. Encyclopedia of Life Sciences.

[B11] Altschul SF, Boguski MS, Gish W, Wootton JC (1994). Issues in searching molecular sequence databases. Nature Genetics.

[B12] Karlin S, Altschul SF (1990). Methods for Assessing the Statistical Significance of Molecular Sequence Features by Using General Scoring Schemes. Proc Natl Acad Sci USA.

[B13] Waterman MS, Vingron M (1994). Rapid and Accurate Estimates of Statistical Significance for Sequence Database Searches. Proc Natl Acad Sci U S A.

[B14] Altschul SF, Gish W (1996). Local Alignment Statistics. Methods in Enzymology.

[B15] Pearson WR (1998). Empirical Statistical Estimates for Sequence Similarity Searches. Journal of Molecular Biology.

[B16] Mott R, Tribe R (1999). Approximate Statistics of Gapped Alignments. Journal of Computational Biology.

[B17] Mott R (2000). Accurate Formula for P-values of Gapped Local Sequence and Profile Alignments. Journal of Molecular Biology.

[B18] Altschul SF, Bundschuh R, Olsen R, Hwa T (2001). The estimation of statistical parameters for local alignment score distributions. Nucleic Acids Research.

[B19] Schäffer AA, Aravind L, Madden TL, Shavirin S, Spouge JL, Wolf YI, Koonin EV, Altschul SF (2001). Improving the Accuracy of PSI-BLAST Protein Database Searches with Composition-based Statistics and Other Refinements. Nucleic Acids Research.

[B20] Sheetlin S, Park Y, Spouge JL (2005). The Gumbel Pre-factor *k *for Gapped Local Alignment can be Estimated From Simulations of Global Alignment. Nucleic Acids Research.

[B21] Yu YK, Gertz EM, Agarwala R, Schäffer AA, Altschul SF (2006). Retrieval Accuracy, Statistical Significance and Compositional Similarity in Protein Sequence Database Searches. Nucleic Acids Research.

[B22] Kschischo M, Lässig M, Yuc YK (2004). Toward an Accurate Statistics of Gapped Alignments. Bulletin of Mathematical Biology.

[B23] Grossmann S, Yakir B (2004). Large Deviations for Global Maxima of Independent Superadditive Processes with Negative Drift and an Application to Optimal Sequence Alignments. Bernoulli.

[B24] Pagni M, Jongeneel CV (2001). Making Sense of Score Statistics for Sequence Alignments. Briefings in Bioinformatics.

[B25] Pearson WR, Wood TC, Balding DJ, Bishop M, Cannings C (2001). Statistical Significance in Biological Sequence Comparison. Handbook of Statistical Genetics.

[B26] Mitrophanov AY, Borodovsky M (2006). Statistical Significance in Biological Sequence Analysis. Briefings in Bioinformatics.

[B27] Agrawal A, Brendel V, Huang X (2008). Pairwise Statistical Significance Versus Database Statistical Significance for Local Alignment of Protein Sequences. Bioinformatics Research and Applications.

[B28] Agrawal A, Brendel V, Huang X (2008). Pairwise Statistical Significance and Empirical Determination of Effective Gap Opening Penalties for Protein Local Sequence Alignment. International Journal of Computational Biology and Drug Design.

[B29] Kotz S, Nadarajah S (2000). Extreme Value Distributions: Theory and Applications.

[B30] Wolfsheimer S, Burghardt B, Hartmann AK (2007). Local Sequence Alignments Statistics: Deviations from Gumbel Statistics in the Rare-event Tail. Algorithms Mol Biol.

[B31] Hartmann AK (2002). Sampling Rare Events: Statistics of Local Sequence Alignments. Physical Review E.

[B32] Olsen R, Bundschuh R, Hwa T (1999). Rapid Assessment of Extremal Statistics for Gapped Local Alignment. Proc of the Seventh International Conference on Intelligent Systems for Molecular Biology.

[B33] Mott RF (1992). Maximum-likelihood Estimation of the Statistical Distribution of SmithWaterman Local Sequence Similarity Scores. Bulletin of Mathematical Biology.

[B34] Sierk ML, Pearson WR (2004). Sensitivity and Selectivity in Protein Structure Comparison. Protein Science.

[B35] Brenner SE (1998). Practical database searching. Trends in Biotechnology.

[B36] Bucher P, Hofmann K (1996). A Sequence Similarity Search Algorithm Based on a Probabilistic Interpretation of an Alignment Scoring System. Proceedings of the Fourth International Conference on Intelligent Systems for Molecular Biology.

[B37] Orengo CA, Michie AD, Jones S, Jones DT, Swindells MB, Thornton JM (1997). CATH – A Hierarchic Classification of Protein Domain Structures. Structure.

[B38] Rocha J, Rosselló F, Segura J (2006). Compression Ratios Based on the Universal Similarity Metric Still Yield Protein Distances far from CATH Distances. CoRR.

